# Convective drying kinetics of faecal sludge from VIP latrines

**DOI:** 10.1016/j.heliyon.2022.e09221

**Published:** 2022-03-29

**Authors:** J. Pocock, S. Septien, B.S.N. Makununika, K.V. Velkushanova, C.A. Buckley

**Affiliations:** a Chemical Engineering Discipline, University of KwaZulu-Natal, 4041, Durban, South Africa; b WASH R&D Centre (formerly known as the Pollution Research Group), University of KwaZulu-Natal, 4041, Durban, South Africa

**Keywords:** Onsite sanitation, Moisture content, Drying curves, Effective diffusivity, Modelling

## Abstract

In striving to achieve sustainable sanitation, one challenge is to ensure hygienic treatment of faecal sludge from on-site sanitation. Thermal drying is an important treatment step for moisture removal and disinfection. Improved understanding of the drying process is crucial for the proper design of treatment technologies for faecal sludge. In this study, faecal sludge from ventilated improved pit (VIP) latrines from Durban, South Africa, were dried in a convective drying thermobalance by varying the temperature from 40 to 80 °C, the relative humidity from 0 to 25% and the air velocity from 0.3 to 1.2 mm/s. The faecal sludge samples were in the form of a thin layer and pellets with different diameters from 8 to 14 mm. Kinetic parameters were determined from the experimental data, were compared to classical drying models in literature and were then used to develop a correlative drying model.

Drying rates ranged between 1 and 40 g/min/m^2^, leading to drying times comprised between 100 and 300 min. The drying kinetics increased as temperature was higher, and pellet diameter and relative humidity were lower. Temperature had the greatest influence on the drying kinetics (in both the constant and falling rate periods), followed by the effect of pellet diameter. The drying kinetics were affected in a moderate way by the relative humidity in the constant rate period and part of the falling rate period. The air velocity had a slight effect of drying kinetics during the constant rate period, but this becomes insignificant during the falling rate period. The effective diffusivities increased from 7.81 × 10^−8^ to 1.97 × 10^−7^ m^2^/s by increasing the temperature from 40° to 80 °C, leading to an activation energy of 23 kJ/mol. These values are typical from those found for wastewater sludge. The sludge exhibited a critical moisture content varying between 2.4 and 3.2 g/g db during drying without a clear trend as a function of the operating conditions and suggested that sludge was composed of considerably more bound moisture than unbound.

The experimental data fitted the most closely to the Page model and, based on this, a new model was proposed for the prediction of drying times across the range of explored temperatures and pellet diameters in this study. The results of the proposed model fitted the experimental data with acceptable accuracy, so that the developed model could be employed as an analytical tool for the design, operation and optimisation of drying equipment.

## Highlights

• Faecal sludge convective drying was studied and modelled.
• Temperature and sample size had the greatest effect on drying.
• Air velocity and relative humidity had low or null effect on drying.
• The *D*_*eff*_ and *E*^*a*^ were in the order of 10^−7^ m^2^/s and 20 kJ/mol respectively.
• The developed model in this work, based on the Page model, presented good accuracy.

## Graphical abstract

**Figure:**
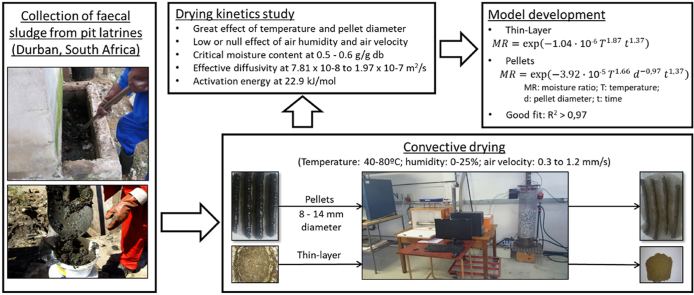

## 1 Introduction

Ensuring hygienic, functional and sustainable sanitation is a global challenge, particularly in developing countries. The provision of improved sanitation was selected as one of the key issues in the United Nations Millennium Development Goals, as well as in the Sustainable Development Goals [1]. According to the World Health Organisation (WHO), an estimated of 2.4 billion people still do not have access to a proper sanitation, with over 946 million people practising open defecation, although considerable progress has been made since 1990 [2]. A major part of the population of the world, particularly in Africa and Asia, rely on on-site sanitation facilities such as septic tanks, pour-flush toilets, pit latrines and ventilated improved pit latrines, as a consequence of the lack of a sewage system.

The “Reinvent the Toilet Challenge” (RTTC) program, initiated by the Bill & Melinda Gates Foundation (BMGF), focuses on the development and implementation of innovative technologies for the processing of human waste from on-site sanitation systems. The main aim of the RTTC is to develop sanitation systems that can integrate and utilise different excreta waste streams and recover valuable constituents such as energy, sterilised fertiliser and potable water. These must have a cost lower than 0.05 $/user/day, not be connected to the grids (water supply, sewerage network or electricity), have little or no net energy usage, and be culturally acceptable [3, 4]. Sanitation practitioners give great importance to resource recovery from innovative faecal sludge treatment processes with the objective to generate revenue that could make sustainable the sanitation chain. Indeed, finding appropriate approaches for resource recovery is a major step towards improving the current sanitation situation. Diener et al. [5] stated that viable business models could emerge if faecal sludge management systems are designed with the goal of resource recovery. Different resource recovery routes have been proposed for faecal sludge management, including the use of the faecal sludge as a biofuel [6], in agriculture [7] and protein derivation for animal feed [8], among others.

Prior to reuse, faecal sludge drying is a critical and necessary treatment process. Thermal drying has been widely employed over the years to dehydrate the sludge from wastewater treatment facilities [9]. Drying reduces the mass and volume of the sludge, leading to the reduction of costs related to its storage, transportation, packaging and further processing. In addition, drying at high temperatures deactivates pathogenic organisms, making the sludge hygienic and safe to handle or use. It also turns the material into a suitable combustible, by increasing the lower calorific value through the decrease of the moisture content.

The understanding, characterisation and modelling of drying kinetics is the first step to develop a reactor model for the design, operation, control and optimisation of drying processes [10, 11, 12]. A kinetic model enables to predict the required residence time to achieve the desired moisture content under a specific set of operating conditions. It constitutes an advantageous tool that can help for process optimization to maximise productivity, and minimise energy consumption and costs. The development of such a model requires kinetic data and understanding about the drying process.

Different types of drying kinetics models can be found in literature, particularly in the food industry sector. The complexity of the models varies widely from simple empirical and semi-theoretical expressions to more complex mechanistic modelling approaches. The empirical and semi-theoretical models consist in mathematical expressions where the parameters can lump several physical phenomena and are determined by mathematical fitting [13, 14]. The mechanistic models are based on the fundamental laws of mass, momentum and energy conservation. They consider, at different level of detail, the phenomena involved on the drying process, such as the transport equations and thermodynamic equilibrium phase, and the changes in the physiochemical properties of the material, such as shrinkage and porosity [10, 15]. The mechanistic models have the capability to predict several phenomena from the drying process, e.g. the heat and mass transfer fluxes, but they may require the determination of parameters difficult to measure and involve mathematical and computational complexity that limits their application [14]. The semi-empirical and empirical models are distinguished by their simplicity even though they cannot give a real representation of the physics of the system, but this may be enough for engineering practical applications.

Information about drying kinetics is lacking for faecal sludge from on-site sanitation. A few studies about human faeces and faecal sludge drying kinetics have been reported in literature [16, 17, 18] but none of these works have modelled their kinetic data. A few models have been developed for sewage sludge drying [19, 20, 21], which could serve as reference for the development of drying models specific to faecal sludge but cannot be directly transposed as the characteristics from sewage and faecal sludge differ in many aspects.

Due to limited kinetic studies in literature, there is the need to generate more data and insight about faecal sludge drying. Moreover, literature does not offer a drying kinetic model developed specifically for faecal sludge, which would be a useful tool for the optimal design and operation of faecal sludge drying technologies. In response to these gaps, the current work aims at generating data, gaining insight and developing a pratical model for feacal sludge drying that is simple to put into application.

Faecal sludge from ventilated improved pit (VIP) latrines was selected as the feedstock, as this type of toilet facility, considered as the minimum acceptable level of sanitation [22, 23], is the most commonly used in sub-Saharan Africa [24]. A customised experimental rig was employed to determine the convective drying kinetics as a function of different operating conditions (air velocity, temperature and relative humidity), from which an empirical kinetic model was developed. Convective (or hot gas) drying was chosen as the preferred type of drying method for this study, as it is widely applied in industry and thus many sanitation technologies would rely on this method. Faecal sludge drying kinetics were studied for two geometries: a thin layer and pellets. In literature, the study of drying kinetics is typically conducted on a thin layer of the material to dry, which offers a simple analysis of the kinetic data that could be extrapolated to more complex geometries. Pellets is an attractive shape for faecal sludge processing that is already done in some processes as the LaDePa in Durban, South Africa [16, 25]. One of the limits of the experimental apparatus in this work is that it could not cover the wide range of the operating conditions from industrial dryers, in particularly regarding the air velocity.

## 2 Material and methods

### 2.1 Feedstock: faecal sludge from VIP latrines

The sample employed in this study was faecal sludge from VIP latrines in the eThekwini municipality (Durban, South Africa). In eThekwini municipality, the VIP latrines are meant to be used mainly for urination and defection events, including the disposal of anal cleansing material that can be either toilet paper or newspaper. Nevertheless, in practice, the users of the latrines also tend to dispose other kind of materials, such as solid waste, greywater and detergents. Therefore, the sludge can be considered as a mixture of urine with faecal material, encompassing water, foreign objects and chemicals (particularly detergents).

The samples were collected during emptying of the VIP latrines located in the peri-urban area of Durban. Pit emptying is commonly performed manually using shades and shovels to remove the sludge, and buckets to transport the emptied sludge to the disposal site. Sampling was conducted by taking a small portion of the removed sludge from the different sections of the pit. This operation was repeated for a few pits. The resulting sample was homogenised through a thorough mixing to get a composite material representative of the involved pits. More details about the sampling procedure are given in a previous study [26].

After sampling, the sludge was kept in a cooler box with ice and transported to the laboratory from the WASH R&D Centre, at the University of KwaZulu-Natal. In the laboratory, the sludge was placed in the cold room at 4 °C until used for experiments, in order to limit or stop any biological degradation and dehydration that can alter the physiochemical properties of the material.

After analysis of the feedtock, the faecal sludge exhibited a moisture content of 80%wt (equivalent to 4 g/g db) and ash content of 50 g/g db, which were representative to the average composition of the VIP latrine sludge in the eThekwini municipality [27].

### 2.2 Convective drying rig

**Figure 1:**
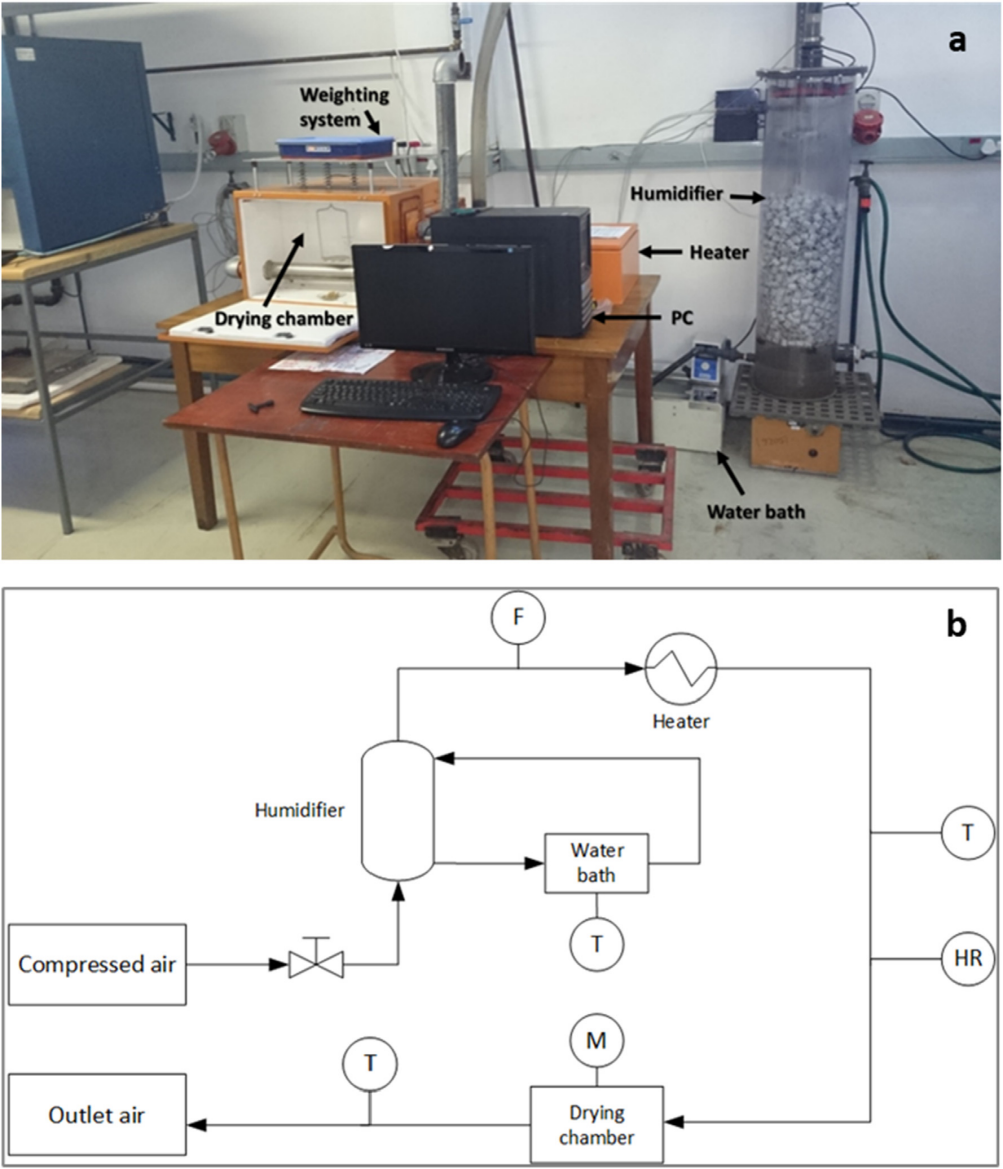
Photograph (a) and schematic diagram (b) of the drying rig (F: flow measurement; T: temperature measurement; M: mass measurement; HR: relative humidity measurement).

A thermobalance was assembled for the drying experiments. This apparatus can be classified as a direct-heat dryer, which provides the energy to heat the material with hot air. It is composed of three components, which are: the humidification section, the heating section, and the drying section. A photograph and schematic diagram of the experimental rig are presented in Figure 1.

Dehumidified air, supplied by a compressor, was employed for the experiments. The volumetric air flowrate was determined by temperature and differential pressure measurements, and was regulated by a globe valve. The humidification section consisted of a packed column with plastic Raschig rigs, where the air stream was introduced through the bottom and put into counter-current contact with water droplets falling from a sprinkler head, located at the top of the column. The absolute humidity of the air used was adjusted by controlling the temperature of the water circulating in the column, based on the assumption that the outlet air from the column was at the water bath temperature.

The humidified air passed then through the heating section, where electric heating coils elevated its temperature to the set value. After heating, the hot air stream moved to the drying chamber where the faecal sludge sample was dried. The drying chamber was constituted of a sample holder, which was suspended on a precision weighing strain gauge load cell with an accuracy of 0.01 g, connected to a computer. The sample mass was measured on-line to track the change in mass with time. The air temperature and relative humidity were monitored at the inlet and outlet of the drying chamber, and the values from the measurements were continually logged on the computer. The interface included a control system to maintain the drying temperature at the set value. The temperature of the water bath and air flowrate were adjusted manually by the experimenter.

### 2.3 Experimental procedure

This work was done under the ethical clearance exemption from the Biomedical Research Ethics Committee from the University of KwaZulu-Natal (EXM005/18).

The experiments were performed for two different forms of the feedstock, these being a thin layer and pellets of varying diameter. Prior to the experiments, the sludge was sieved using a 5 mm grid in order to remove detritus such as plastics and textiles, and was allowed to reach room temperature.

The pellets were produced by extrusion using a hand-held extruder, composed of a 64 mm diameter PVC pipe with an air inlet with a ball valve in one end and a nozzle at the other end. During preparation for extrusion, the pipe was filled with sludge and connected to the compressor airline. Then, the valve was opened to move the sludge out or the pipe through the nozzle by the force exerted by the compressed air, leading to the formation of pellets. The diameter of the pellets was varied by changing the extruder nozzle size. For the thin layer geometry, faecal sludge was placed in a cylindrical crucible with a 70 mm diameter and height of 7 mm. The initial mass of the sample varied between 30 to 50 g, depending on the type of sample.

Before starting the drying experiments, the experimental rig was run at the selected operating conditions without sample for approximately 30 min, so as to ensure that steady conditions were achieved. During the experiments, the sample mass was recorded at intervals of 5 min and the test was stopped 30 min after mass stabilisation.

The following conditions were investigated during the faecal sludge drying tests:

• Temperatures of 40, 50, 60, 70 and 80 °C (controlled by the air heater);
• Relative humidities (RH) of 5, 15 and 25% (determined from the drying temperature and the absolute humidity obtained after humidification of the air at a given temperature in the packed column);
• Air velocities of 0.3, 0.6 and 1.2 mm/s in the drying chamber (determined from the air flowrate introduced into the system and the cross section area of the drying chamber);
• Pellet diameters of 8, 10, 12 and 14 mm (only for pellets).

Table 1 summarises the experiments conducted in this investigation. Each of the experiments was repeated two or three times, in order to evaluate the repeatability of the results and to determine the statistical variation. In general, the results presented a good reproducibility.

**Table 1:** Summary of the experiments performed for the thin layer samples and pellets.

T (°C)	RH (%)	Air velocity (mm/s)	Thin layer	Pellets			
				8 mm	10 mm	12 mm	14 mm
40	5	0.6	X	X			
	15	0.6			X		
	25	0.6			X		
50	5	0.6	X				
60	5	0.3	X		X		
		0.6	X	X	X	X	X
		1.2	X		X		
	15	0.6	X	X	X		
	25	0.6	X	X	X		
70	5	0.6	X				
80	5	0.6	X	X	X		X
	15	0.6			X		

### 2.4 Data analysis

#### 2.4.1 Drying curves

During the drying experiments, the sample mass was constantly monitored and logged on the computer. From these measurements, the drying curve, representing the variation of the moisture content versus time, was plotted for each experiment. This was based on the assumption that the mass loss during the experiments was exclusively due to moisture evaporation.

The moisture content at a given instant was calculated from Eq. (1).

(1) $$x_{i}=\frac{m_{i}-m_{s}}{m_{s}}$$

With $x_{i}$ the moisture content in dry basis at the instant i [g/g db], $\;m_{i}$ the mass of sample at the instant i [g] and $m_{s}$ the mass of solids [g].

The mass of solids was calculated by multiplying the initial mass of sample by the total solid content of the sludge, which represented 20%wt from measurements.

#### 2.4.2 Drying rate curves

The drying rate, which refers to the rate at which moisture is evaporated over time, was calculated by dividing the differential of the sample mass between two subsequent measurements by the elapsed time, and by the initial surface area of the sample (assumed to remain constant along the transformation as approximation). Eq. (2) depicts the formulae employed to calculate the drying rate at any instant.

(2) $${\dot{m}}_{i}=\frac{m_{i}-m_{i+1}}{\left(t_{i}-t_{i+1}\right)}\times \frac{1}{S}$$

With ${\dot{m}}_{i}$ the drying rate at the instant i [g/min/m^2^], $t_{i}$ and $t_{i+1}$ the time at the instant i and (i+1) respectively [min], $m_{i}$ and $m_{i+1}$ the sample mass at the instant i and (i+1) respectively [g], and *S* the surface area of the sample [m^2^].

Prior to the calculation of the drying rate, the mass measurements over time were smoothed to eliminate the noise within the data, by using a polynomial function fit over a number of adjacent data points as stipulated by Kemp et al. [28].

#### 2.4.3 Effective moisture diffusivity

The internal transfer rate of moisture within the sample during drying was evaluated through the effective moisture diffusivity, which lumps various mass transfer phenomena, such as: liquid and vapour diffusion, moisture movement due to liquid capillary suction forces and static pressure differences, among other phenomena. The thin layer geometry was used to determine the effective moisture diffusivity as this required a simpler mathematical approach. The thin layer samples were thereby considered as a thin slab with one surface exposed to the air.

The effective moisture diffusivity is typically calculated during the falling rate period using Eq. (3), which is derived from Fick's Law and is valid for a thin plate geometry [29]. This equation is based on the fallowing assumptions:

(i) The sludge is a homogeneous mixture with uniform moisture distribution.
(ii) The movement of moisture due to thermal gradient within the sample is negligible so that moisture mass transfer is regarded as a one-dimensional diffusion process.
(iii) The shrinkage of the sludge is negligible during drying.

(3) $$MR\left(t\right)=\frac{x\left(t\right)-x_{eq}}{x\left(t=0\right)-x_{eq}}=\frac{8}{\pi^{2}}\overset{\infty }{\underset{n=1}{\sum }}\frac{1}{{\left(2n-1\right)}^{2}}\exp\left[-{\left(2n-1\right)}^{2}\frac{\pi^{2}D_{eff}t}{4z^{2}}\right]$$

With MR(t) the moisture ratio, x(t) the moisture content at time t [g/g], $x_{eq}$ the moisture content at the thermodynamic equilibrium [g/g], x(t=0) the initial moisture content [g/g], $D_{eff}$ the effective diffusivity [m^2^/s] and z the half thickness of the slab [m]. The equilibrium moisture content was considered as the final value achieved by the sample after the stabilization of its mass during drying. Eq. (3) can be simplified by considering only the first term of the summation series solution and then linearising the expression, which leads to Eq. (4).

(4) $$ln\left(MR\left(t\right)\right)=ln\left(\frac{8}{\pi^{2}}\right)-\frac{\pi^{2}D_{eff}t}{4z^{2}}$$

The effective moisture diffusivity can be determined through the value of the gradient of the plot ln(MR(t)) versus t. The relationship of the effective diffusivity with temperature, represented by Arrhenius law in Eq. (5), was determined by varying the temperature between 40 and 80 °C whilst holding all other variables constant.

(5) $$D_{eff}=D_{0}\;\exp\left[-\frac{E_{a}}{RT}\right]$$

With $D_{0}$ the pre-exponential factor [m^2^/s], $E_{a}$ the activation energy [J/mol] and R the ideal gas constant [8.3145 J/mol].

The activation energy and pre-exponential factor can be deduced by linearising Eq. (5) into Eq. (6) and plotting $\ln\left(D_{eff}\right)$ versus *(*$\frac{1}{T}$). The slope of the line corresponds to $\frac{E_{a}}{R}$ and the intercept with the origin equals to $\ln\left(D_{0}\right)$.

(6) $$\ln\left(D_{eff}\right)=\ln\left(D_{0}\right)-\frac{E_{a}}{RT}$$

#### 2.4.4 Modelling

Various empirical classical drying models were tested on the experimental results: the Newton model (Eq. (7)) [30], the Page model (Eq. (8)) [31], the Modified Page model (Eq. (9)) [32], the Approximation of Diffusion model (Eq. (10)) [33] and the Handerson and Pabis model (Eq. (11)) [34]. These models were selected as they have proven to represent well the drying kinetics of sludges.

(7) MR=exp(−kt)

(8) $$MR=\exp\left(-kt^{n}\right)$$

(9) $$MR=\exp\left(-{\left(kt\right)}^{n}\right)$$

(10) MR=a ​exp(−kt)+(1−a)exp(−kat)

(11) MR=a ​exp(−kt)

With *a*, *k* and *n* the parameters from the models. An optimisation tool, SOLVER (GRG2 method), included in Microsoft Excel, was used for the determination of the models parameters by regression, i.e. by minimising the sum of the square differences between the experimental and the calculated values.

The results from the model were compared to the experimental data by using the goodness of fit statistical measure. This entails the determination of the coefficient of determination $R^{2}$ (Eq. (12)), which evaluates how well the model fits to the experimental data, and the root mean square error RMSE (Eq. (13)), which reflects the dispersion of the experimental and modelling data. The accuracy of the model is higher as *R*^*2*^ is close to 1 and *RMSE* close to 0.

(12) $$R^{2}=\frac{\sum_{i=1}^{N}{\left(MR_{model,i}-{\bar{MR}}_{exp}\right)}^{2}}{\sum_{i=1}^{N}{\left(MR_{exp,i}-{\bar{MR}}_{exp}\right)}^{2}}$$

(13) $$RMSE=\sqrt{\frac{\sum_{i=1}^{N}{\left(MR_{exp,i}-MR_{model,i}\right)}^{2}}{N}}$$

With ${\bar{MR}}_{exp,i}$ the average experimental moisture ratio, $MR_{exp,i}$ and $MR_{model,i}$ the experimental and modelling moisture ratio at point i respectively, and N the total number of points.

## 3 Results and discussion

### 3.1 Analysis of faecal sludge drying kinetics

**Figure 2:**
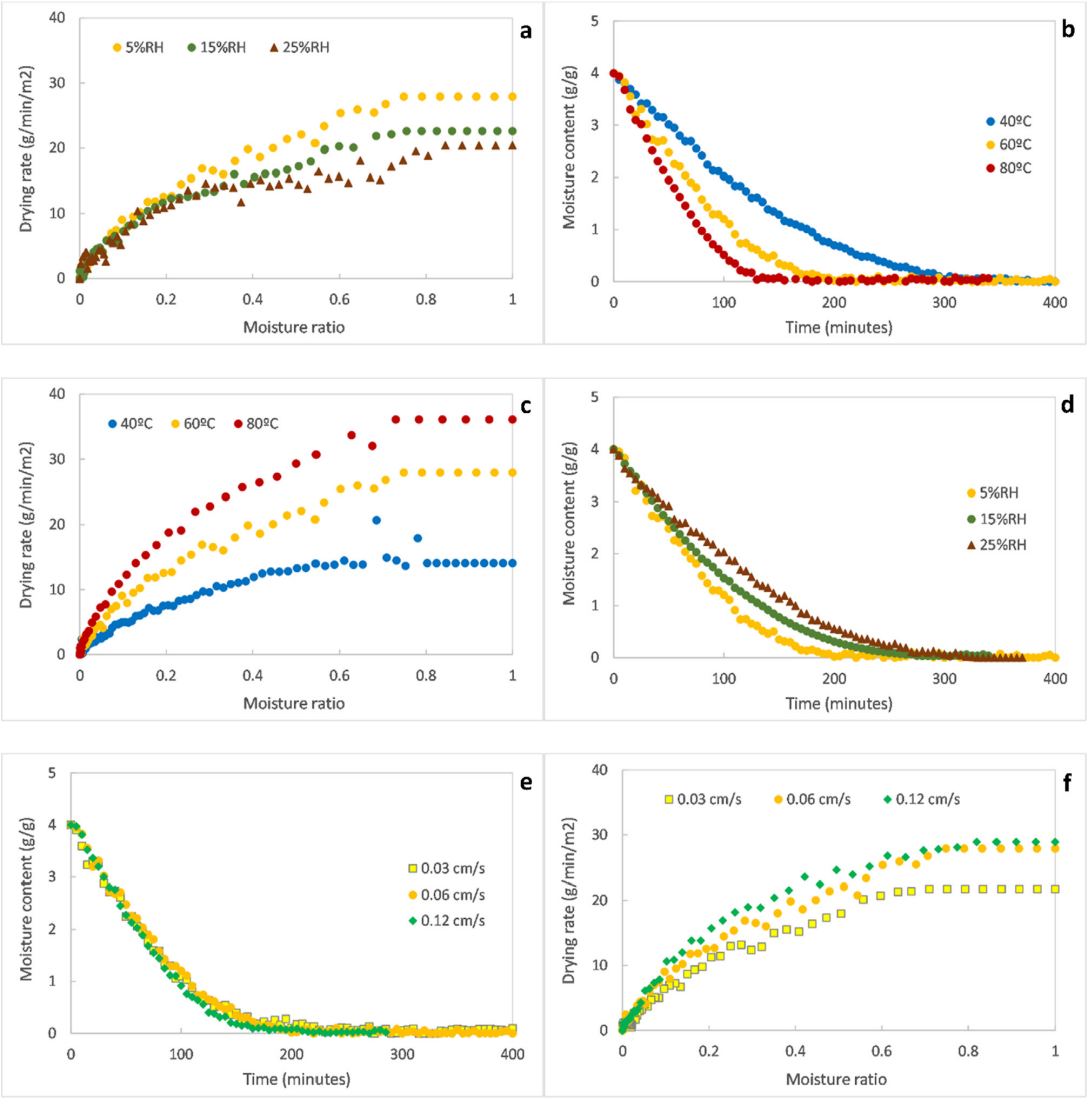
Drying curves and drying rates as a function of temperature at 5% RH and air velocity of 0.6 mm/s (a, b); as a function of relative humidity at 60 °C and air velocity of 0.6 mm/s (c, d); as a function of air velocity at 60 °C and 5% RH (e,f).

**Figure 3:**
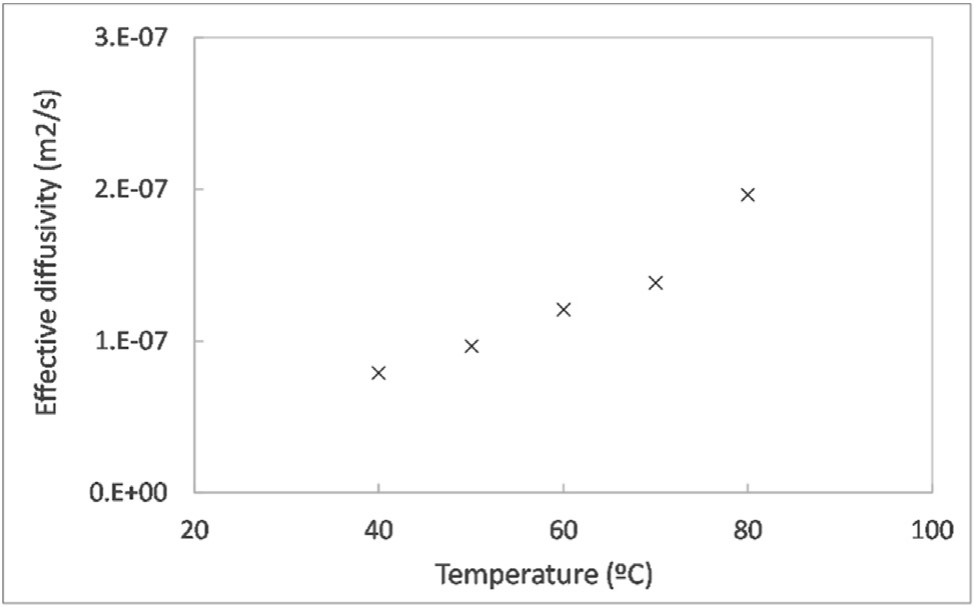
Effective diffusivity as a function of temperature during the thin layer drying at 5% RH and 0.6 mm/s air velocity.

**Figure 4:**
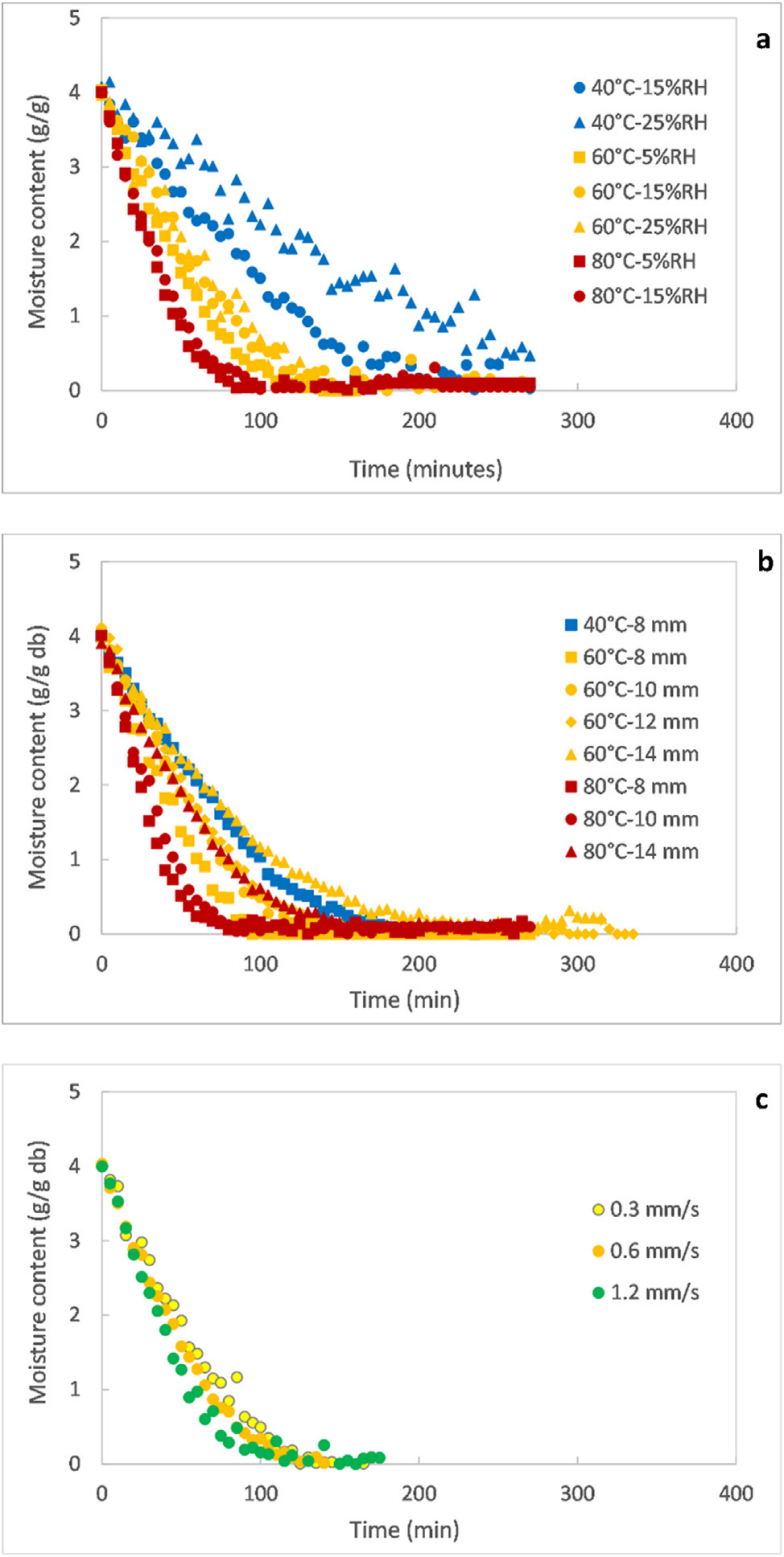
Drying curves as a function of temperature and relative humidity at an air velocity of 0.6 mm/s for the 10 mm pellet (a); as a function of temperature and pellet diameter at 5% RH and air velocity of 0. 6 mm/s (b); as a function of air velocity at 60 °C and 5% RH for the 10 mm pellets (c).

In this section, the drying kinetics are first studied for the thin layer as a function of the air temperature, relative humidity and air velocity. This study comprises the analysis of the drying curves and drying rate curves in Figure 2 and the effective diffusivity in Figure 3. Thereafter, the drying kinetics are studied for the pellets as a function of the operating conditions and the pellet diameter in Figure 4.

#### 3.1.1 Drying kinetics of a thin layer

Figure 2a and b show the variation of moisture content with time and drying rate with moisture ratio as a function of temperature. The two typical convective drying regimes were observed in the graphs, i.e. the constant and falling rate periods. As expected, the time for moisture removal was reduced as the air was heated at a higher temperature, due to faster drying rates during both constant and falling rate periods. This result could be expected since the air had an almost null relative humidity. The moisture content was approximately equal to 0 g/g at the end of the experiments, suggesting that the pellets were bone-dried. This result could be expected since the air had an almost null relative humidity. The critical moisture ratio (i.e. transition between the constant and falling rate period) varied between 0.6 and 0.8 (equivalent to a moisture content range of 2.4–3.2 g/g db), without a clear trend with respect to temperature.

The influence of temperature on the constant and falling rate periods can be explained by its influence on the mass and heat transfers, supposing that the kinetics during the constant rate period are controlled by the external transfers while the falling rate period is mostly controlled by the internal transfer. Thereby, an increase of air temperature is expected to speed the process by increasing the temperature gradients between the material and its surroundings and the mobility of moisture, leading to higher heat and mass transfer rates, in addition to augmenting the amount of heat available for moisture evaporation.

In order to study the effect of temperature on the internal mass transfer into more detail, the effective diffusivity was calculated from the kinetic data during the falling rate period, following the method described in section 2.4.3. The results from this analysis, summarised in Figure 3, demonstrate that the effective diffusivity increased by an order of magnitude by increasing the temperature from 40 °C to 80 °C. The effective diffusivities varied between 7.9 to 19.7 × 10^−8^ m^2^/s. The effective diffusivities found in literature for wastewater sludge dried by different methods, including solar drying [35, 36, 37], contact drying [38], convective drying [39, 40, 41] and drying by electromagnetic induction heating [42] were in the order of 10^−11^ to 10^−7^ m^2^/s. This result denoted that faecal sludge had similar or higher effective diffusivities than wastewater sludge.

From the relationship between effective diffusivity and temperature, the activation energy was calculated as 23 kJ/mol based on the method described in section 2.4.3. This value is in the order of magnitude of what could be expected for a process controlled by transfer phenomena, and is similar to those reported for other types of faecal sludge and fresh faeces (∼30 kJ/mol) [43], and wastewater sludges (10–30 kJ/mol) [35, 38, 39, 40].

Figure 2c and d display the effect of varying the humidity of the heated air at 60 °C on drying. The sludge was almost completely dried in the three experimental cases, even in an atmosphere at 25% relative humidity where some moisture could be expected in the sludge. It can be deduced then that, under the explored conditions, complete drying could be achieved at the thermodynamic equilibrium for a relative humidity below 25%, implying an equilibrium moisture content equal or close to 0.

Similarly to temperature, the relative humidity had an influence on the drying kinetics of the process, but its effect was less pronounced. As could be expected, an increase of humidity in the air led to longer drying times by lowering the drying rates. The time for the complete moisture removal varied between 200 min in a relative dry atmosphere (5%RH) and 300 min in humid air (15 and 25% RH). During the constant rate period, an increase of the relative humidity from 5 to 25% decreased the drying rate from 28 to 20 g/min/m^2^, representing a reduction of approximately 30%. This difference was attenuated during the falling rate period progressed, and eventually disappeared at moisture ratio of 0.3 (corresponding to a moisture content of approximately 1.0 g/g db). No effect of air humidity on the critical moisture content was observed under the explored conditions.

The air humidity influence on the drying rate during the constant rate period and beginning of the falling rate period is in agreement with the theory, whereby a decrease in the relative humidity is expected to increase the moisture concentration gradient between the sludge and surrounding air, leading to an increase of the mass transfer driving force and then to a subsequently increased moisture evaporation rate. This affects mostly the external mass transfer, i.e. the transfer of moisture from the surface of the sludge towards the environment. As the external transfer rates control the constant rate period and influence the falling rate on its early stage to a certain extent, the air humidity should have only an influence on the drying kinetics on these stages, as corroborated in Figure 2d.

The drying curves and drying rate curves as a function of the air velocity can be seen in Figure 2e and f respectively. The variation of the air velocity had a minimal effect on the drying kinetics. At the constant rate period, an increase of the air velocity from 0.3 to 0.6 mm/s caused a moderate increase of the drying rate from 22 to 28 g/min/m^2^, representing an increment of nearly 35%. However, a further increase of air velocity to 1.2 mm/s was not translated into any significant further increase. The drying rate diffence between the different air velocities was low across the falling rate period, with drying at 1.2 mm/s presenting the highest rates by a slight margin, followed by drying at 0.6 mm/s and finally at 0.3 mm/s.

#### 3.1.2 Drying kinetics of pellets

Figure 4a displays the combined effect of temperature and relative humidity on the moisture content evolution during drying of the 10 mm pellets. It can be observed that the drying curves were mainly influenced by temperature while the air relative humidity had a minor influence. The pellets dried the fastest at 80 °C and the slowest at 40 °C, with drying at 60 °C being intermediary between these last two temperatures, regardless of the air relative humidity. Indeed, the pellets required more than 200 min for complete drying at 40 °C whereas this time was shortened to 100 min at 80 °C, reflecting a drying time reduction by more than the half by a temperature increment of 40 Cº. The effect of the relativity humidity on the drying kinetics depended on temperature and diminished as temperature was higher. At 40 °C, the influence of the air humidity could be clearly seen on the drying kinetics, with a lower humidity leading to faster drying. This difference became smaller at 60 °C and was no longer noticeable at 80 °C.

Figure 4b shows the combined effect of temperature and pellet diameter on the drying curves. As expected, the drying of pellets was faster as the pellet diameter was lower or the air temperature higher. The fastest drying rate was achieved for the pellets of lowest diameter (8 mm) exposed to the highest temperature (80 °C). It could be also observed that there was no dominant effect on the drying kinetics between the temperature and pellet size. This entails that the pellets did not dry faster only because the temperature was higher, but the pellet diameter had to be considered as well. For example, the 10 mm pellets dried faster at 40 °C compared to the 14 mm pellets at 60 °C. A similar case was observed for the 14 mm pellets that dried at a lower rate at 80 °C compared to the 8 mm pellets drying at 60 °C, or at a comparable rate to the 10 and 12 mm pellets drying at 60 °C.

Considering the effect of pellet size on the drying kinetics alone, the required time to dry the sludge at a fixed temperature was importantly reduced by decreasing the diameter. For instance, the reduction of the diameter from 14 to 8 mm allowed shortening the drying time from 170 to 80 min at 60 °C, and from 230 to 100 min at 80 °C, representing a drying time reduction in the order of 100 min for a 6 mm reduction of diameter. Indeed, the decrease of the pellet diameter offers a lower resistance to the heat and mass transfer with the surrounding air by decreasing the boundary layer thickness at the surface of the material. It also shortens the distance of heat propagation and moisture migration within the material, subsequently leading to faster internal heat and mass transfer.

As it can be seen in Figure 4c, the air velocity did not yield to any remarkable variation of the drying kinetics. The process occurred at a slightly faster rate with increasing air velocity, as also found for the thin layer experiments. The minimal effect of air velocity could be caused by a too narrow range of tested air velocities in this study that precluded a meaningful impact on the heat and mass transfer.

#### 3.1.3 Summary

Temperature had the greatest effect on drying kinetics among the airflow operating conditions. An increase of air temperature allowed reduction of the drying time due to faster drying rates in both the constant and falling rate periods. The influence of temperature on the falling rate period was reflected by the increasing effective diffusivity values from 7.81 · 10^−8^ m^2^/s at 40 °C to 19.7 · 10^−8^ m^2^/s at 80 °C, with an activation energy of 23 kJ/mol. The effect of air humidity was moderate in comparison to temperature. Drying tended to be faster as the air was less humid, leading to lower drying times. Air humidity had an influence particularly during the constant rate period and its influence was gradually reduced during the falling rate period. With respect to the air velocity, its effect on the drying kinetics was weak, probably due to the narrow range of air velocities considered in this study. In the case of pellets, the diameter had an influence on the drying kinetics with a comparable influence to temperature.

The operating conditions did not show any influence on the critical moisture content, varying between 2.4 and 3.2 g/g db. Some authors believe that the critical moisture content marks the limit between unbound and bound moisture, based on the assumption that unbound moisture is removed during the constant rate period while the removal of the unbound moisture occurs during the falling rate period [44, 45, 46]. The amounts of unbound and bound moisture can be subsequently quantified from the determination of the critical moisture content. Nevertheless, this assumption is debatable since the operating conditions can also influence the critical moisture content [47]. In our case, the transition from the constant rate period to the falling rate period occurred approximately within the same range of moisture content at the various operating conditions, suggesting that the critical moisture content was an intrinsic property of the sludge that could be related to its moisture boundness properties. It can be noted that the falling rate period was considerably longer than the constant rate period, which could signify that the amount of bound moisture was higher than the amount of unbound moisture. This hypothesis could be corroborated by the viscous aspect of the sludge but requires further verification.

### 3.2 Faecal sludge convective drying modelling

The first step towards the development of a model was to test common semi-empirical models from literature against the drying kinetic data obtained in this study. The model that fitted the best with the experimental data was then selected for the development of a faecal sludge convective drying model. Thereafter, the parameters from the selected model were correlated to the temperature in the case of the thin layer, and temperature and diameter in the case of the pellets. Finally, the developed model was confronted to experimental data, and the application of the model in real cases discussed.

#### 3.2.1 Comparison of models from literature

The kinetic data obtained from the thin layer and pellet drying was fitted to some common empirical models. These models where chosen because they are regarded as the most accurate in describing various food material as well as sludge from wastewater treatment [48, 49, 50]. Table 2 shows the model and goodness of fit parameters for the thin layer drying at different temperatures.

**Table 2:** Parameters of the models from literature regressed from the experimental data, for the thin layer drying experiments at 40, 60 and 80 °C, at 5% RH and an air velocity of 0.6 mm/s.

Model	Temperature	Parameters			*R*^*2*^	*RMSE*
		*n*	*a*	*k*		
Newton	40 °C	-	-	0.00752	0.968	0.052
	60 °C	-	-	0.0125	0.975	0.047
	80 °C	-	-	0.0169	0.971	0.052
Page	40 °C	1.40	-	0.00708	0.999	0.011
	60 °C	1.33	-	0.0118	0.997	0.017
	80 °C	1.37	-	0.0159	0.996	0.018
Modified Page	40 °C	1.40	-	0.000963	0.999	0.011
	60 °C	1.33	-	0.00267	0.997	0.017
	80 °C	1.37	-	0.00342	0.996	0.018
Henderson and Pabis	40 °C	-	1.12	0.00833	0.980	0.041
	60 °C	-	1.09	0.0136	0.983	0.040
	80 °C	-	1.10	0.0184	0.979	0.044
Approximation of Diffusion model	40 °C	-	1.93	0.0111	0.996	0.017
	60 °C	-	1.86	0.0178	0.995	0.021
	80 °C	-	1.89	0.0244	0.994	0.024

According to Table 2, all the models provided a good fit with *R*^*2*^ higher than 0.96 and *RMSE* lower than 0.06. The Page and Modified Page models fitted with the highest accuracy to the experimental data. Although the values reported in the table for these models are identical, the Page model showed a better fit to both geometries, the thin layer and pellets, therefore it was selected for the development of the model in this study. The Page model has previously been reported to be a good fit for drying of foodstuffs [51], wastewater sludge and tomato waste sludge [50, 52].

#### 3.2.2 Development of a convective drying model for faecal sludge

A kinetic model was developed in order to predict the evolution of the moisture content of faecal sludge during drying. The proposed model was based on the Page Model that demonstrated the best fit to the experimental data among the different options, as discussed in the previous section. Temperature and pellet size were taken into account in the model, as they were shown to be the parameters with major influence on the drying kinetics according to the experimental results. The model did not include the effect of air relative humidity nor velocity, as these parameters exhibited a moderate or slight effect on the drying kinetics under the explored conditions, in addition to that the developed model wanted to be kept as simple as possible.

The parameter *k* from the Page Model was correlated as a function of temperature for the thin layer (Eq. (14)), and temperature and diameter for the pellets (Eq. (15)).

(14) $$k=A\;T^{w}$$

(15) $$k=B\;T^{y}d^{z}$$

Where A, B, w, y and z are the model constants, T the drying temperature [°C] and d the pellet diameter [mm]. The model constants were calculated from multiple non-linear regression analyses from the experimental results, yielding to values of 1.04 × 10^−6^, 3.92 × 10^−5^, 1.87, 1.66 and -0.97 for A, B, w, y and z, respectively. It can be noted that the values of the exponent w was slightly higher than that from y, denoting a slightly more important influence of temperature on the case of the thin layer than the pellets.

Interestingly, the parameter *n* remained constant at a value of 1.4 or thereabouts at the different tested conditions (Table 1). An average value of 1.37 was selected for *n* for both thin layer and pellets drying.

#### 3.2.3 Validation of the model

**Figure 5:**
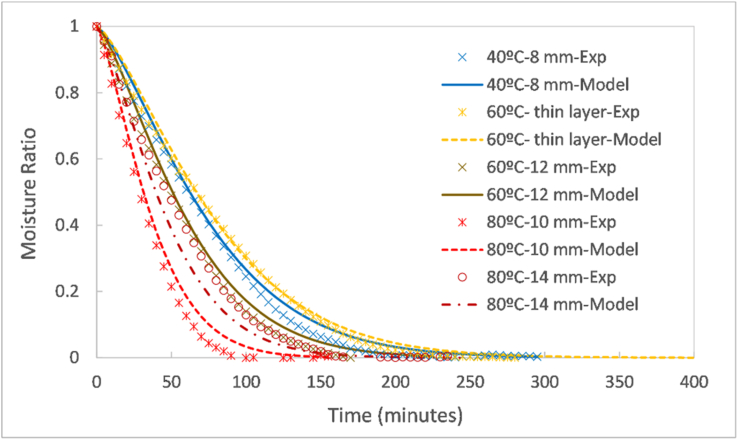
Comparison between the drying curves from the developed model and experiments for a few experimental cases.

The model was confronted with experimental data in order to evaluate its accuracy. In Figure 5, the drying curves computed by the model were compared to the experimental curves from different tests at varying temperature and sample type across the tested range, at constant relative humidity (5%) and air velocity (0.6 mm/s). For each case, the statistical evaluation of the goodness of fit was determined through the *R*^*2*^ and *RMSE*, as displayed in Table 3. Note that the experimental data from the 10 and 14 mm pellets drying at 80 °C did not contribute to the development of the model, whilst the other experimental cases did.

**Table 3:** Statistical analysis of the goodness of fit of the developed model with respect to the data from a few experimental cases.

Experiment		*R*^*2*^	*RMSE*
Temperature	Sample		
40 °C	8 mm pellets	0.993	0.0247
60 °C	Thin layer	0.996	0.0181
	12 mm pellets	0.992	0.0287
80 °C	10 mm pellets	0.985	0.0379
	14 mm pellets	0.981	0.0416

In Figure 5, it can be seen that the modelling and experimental curves were superimposed or separated by a small gap, except for the 14 mm pellets drying at 80 °C where the model underestimated the drying rate. Nonetheless, even in this case, the fit of the model with the experimental data remained reasonable, as can be verified by the high *R*^*2*^ values (>0.97) and low *RMSE* values (<0.05) in Table 3.

In brief, the model accuracy was validated not only from the experimental data that was used for its development, but also from data that was not. The model therefore presents good prediction capabilities.

#### 3.2.4 Application of the model

The developed kinetic model could be inserted in a reactor model for the design of equipment, process control operations and optimization. The model could predict the moisture content of the sludge as a function of time inside a dryer at given operating conditions. This could be particularly useful for the determination of the residence time required to attain a set moisture content.

**Figure 6:**
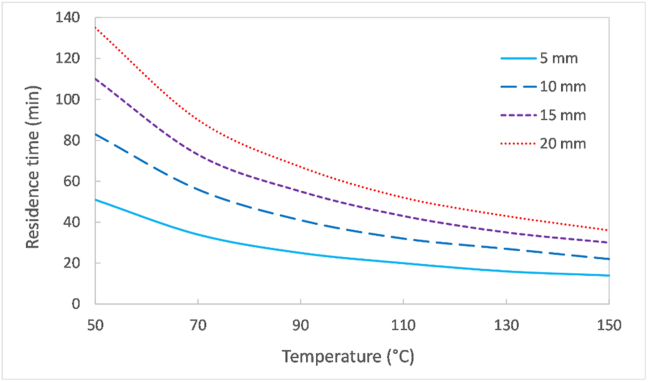
Residence time computed from the model to dry faecal sludge from 80 to 30%wt moisture content as a function of the air temperature and pellet diameter.

Considering a hypothetical example of a drying process with the target to reduce the moisture content of faecal sludge from its initial value, namely 80%wt (4 g/g db), to 30% (0.4 g/g db). The dryer can operate at a temperature ranging between 50 to 150 °C and an extruder can produce pellets from 5 to 20 mm to feed the reactor. The model developed in this study was used to simulate the residence time to achieve the moisture content target as a function of temperature and the pellet diameter. The results from this simulation, presented in Figure 6, indicate that the residence time decreased by increasing temperature and decreasing pellet diameter, as would have been envisaged. Nonetheless, the gradient from the curves diminished by increasing temperature. Therefore, an increase of temperature from a certain point in the dryer would not be worthwhile if considering the energy consumed to heat the air. For instance, the curve corresponding to the 5 mm pellets in Figure 6 starts to flatten at around 100 °C, suggesting that the optimal temperature in this case should be located at the vicinity of 100 °C or even at a lower value. Furthermore, the simulation highlights the importance of operating the dryer with pellets of low diameter, particularly at the low drying temperature region where the difference in pellets size can result in a considerable difference in residence time. For example, drying sludge to 30%wt moisture content at 50 °C would require around half an hour for the 5 mm pellets, whereas this would take more than two hours for the 20 mm pellets. The production of small size pellets would depend on the extruder ability. In the case that this is technically too challenging and the formation of large pellets would be simpler, high temperatures should be employed to achieve fair drying times.

#### 3.2.5 Advantages of the model and limitations

The example above illustrates a possible way to put into application the model for drying process optimisation. The model presents an original approach based on the correlation of the parameters of classical drying models as a function of the operating conditions. To the best of the authors knowledge, this type of model can be found in the food drying field [14], but it has not be reported for sludges. The main advantage of the model is its simplicity to be integrated to a reactor model or process simulation software.

The model can be used with confidence to get accurate predictions in the range of the conditions in which it was developed. Nevertheless, outside of this range, its accuracy cannot be guaranteed, even though it can still provide plausible approximations. The more the model is taken away from the conditions in which it was developed, the higher the possibility of inaccurate predictions, as other events could occur during drying and change the pattern from the trends. Moreover, the fact that the model does not include the effect of air velocity and humidity could cause a deviation from reality under certain circumstances. On the contrary to what was observed in this work, high air velocities as found in industrial convective dryers could have a significant effect on the drying kinetics and the model would not be able to take into consideration this variation. In the case of air humidity, the accuracy of the model simulations could be compromised in some extent at low temperatures (∼40 °C) and humid atmospheres, where the relative humidity can have some influence on the drying kinetics as revealed in Figure 4b, but this should not be a major limitation at higher temperatures (>60 °C). Another limitation of the model is that it was developed from one type of faecal sludge, and it may not take into account the great variability of faecal sludge physiochemical characteristics according of its origin. The model might then not be valid with sludge from other types of on-site sanitation facilities or geographical location.

Further work should be done to verify the accuracy of the model for wider range of operating conditions.

## 4 Conclusions

Air temperature and sample size (here pellet diameter) were revealed as the parameters with a major influence on faecal sludge convective drying kinetics. Relative humidity had a moderate influence on the drying kinetics, mostly at the lower values of the temperature range, and the air velocity was the least influencing parameters, having a slight effect only during the constant rate period. The critical moisture content varied between 2.4 and 3.2 g/g db without following a particular trend as a function of the operating conditions. The value of the critical moisture content presumed a higher amount of bound moisture than that of unbound moisture.

A new model based on the Page model was developed for the prediction of drying kinetics as a function of temperature and pellet diameter. The developed model exhibited an acceptable fit with respect to the experimental data, so it could be employed for the prediction of faecal sludge drying kinetics and could be inserted into a reactor model for process design, control and optimization, but with certain precautions. In future work, the validity of the model at a wider range of operating conditions or with faecal sludge from different sources should be tested.

## Declarations

### Author contribution statement

Pocock, J.: Analyzed and interpreted the data; Wrote the paper.

Septien, S.: Conceived and designed the experiments; Analyzed and interpreted the data; Contributed reagents, materials, analysis tools or data; Wrote the paper.

Makununika, B.S.N.: Performed the experiments; Analyzed and interpreted the data; Contributed reagents, materials, analysis tools or data.

Velkushanova, K.V., Buckley, C.A.: Conceived and designed the experiments.

### Funding statement

This work was supported by the 10.13039/100000865Bill and Melinda Gates Foundation (OPP1069575).

### Data availability statement

Data included in article/supplementary material/referenced in article.

### Declaration of interests statement

The authors declare no conflict of interest.

### Additional information

No additional information is available for this paper.
